# Acoustic-responsive carbon dioxide-loaded liposomes for efficient drug release

**DOI:** 10.1016/j.ultsonch.2023.106326

**Published:** 2023-02-11

**Authors:** Yasuhiko Orita, Susumu Shimanuki, Satoshi Okada, Kentaro Nakamura, Hiroyuki Nakamura, Yoshitaka Kitamoto, Yusuke Shimoyama, Yuta Kurashina

**Affiliations:** a Department of Chemical Science and Engineering, School of Materials and Chemical Technology, Tokyo Institute of Technology, 2-12-1 Ookayama, Meguro-Ku, Tokyo 152-8550, Japan; b Department of Materials Science and Engineering, School of Materials and Chemical Technology, Tokyo Institute of Technology, 4259 Nagatsutacho, Midori-Ku, Yokohama 226-8503, Japan; c Laboratory for Chemistry and Life Science, Institute of Innovative Research, Tokyo Institute of Technology, 4259 Nagatsutacho, Midori- Ku, Yokohama 226-8503, Japan; d Laboratory for Future Interdisciplinary Research of Science and Technology, Institute of Innovative Research, Tokyo Institute of Technology, 4259 Nagatsutacho, Midori- Ku, Yokohama 226-8503, Japan; e Division of Advanced Mechanical Systems Engineering, Institute of Engineering, Tokyo University of Agriculture and Technology, 2-24-16 Nakacho, Koganei-Shi, Tokyo 184-8588, Japan

**Keywords:** **scCO_2_**, supercritical CO_2_, **MEA**, monoethanolamine, **5,6CF**, 5(6)-carboxyfluorescein, **HPLC**, high-performance liquid chromatography, **EE**, Encapsulation efficiency, **MI**, mechanical index, **MRI**, magnetic resonance imaging, **HIFU**, high intensity focused ultrasound, Drug delivery system, Acoustic-responsive material, Ultrasound, Supercritical carbon dioxide, Liposome

## Abstract

The role of liposomes as drug carriers has been investigated. Ultrasound-based drug release methods have been developed for on-demand drug delivery. However, the acoustic responses of current liposome carriers result in low drug release efficiency. In this study, CO_2_-loaded liposomes were synthesized under high pressure from supercritical CO_2_ and irradiated with ultrasound at 237 kHz to demonstrate their superior acoustic responsiveness. When liposomes containing fluorescent drug models were irradiated with ultrasound under acoustic pressure conditions that are safe for the human body, CO_2_-loaded liposomes synthesized using supercritical CO_2_ had 17.1 times higher release efficiency than liposomes synthesized using the conventional Bangham method. In particular, the release efficiency of CO_2_-loaded liposomes synthesized using supercritical CO_2_ and monoethanolamine was 19.8 times higher than liposomes synthesized using the conventional Bangham method. These findings on the release efficiency of acoustic-responsive liposomes suggest an alternative liposome synthesis strategy for on-demand release of drugs by ultrasound irradiation in future therapies.

## Highlights

• CO_2_-loaded liposomes were generated for ultrasonic controlled release of drugs.
• The acoustic responses of liposomes physically loaded with CO_2_ was improved.
• Chemically doping liposomes with monoethanolamine improves acoustic responses.

## 1 Introduction

Liposomes, which are spherical vesicles with lipid bilayers, have been used as transport vehicles to deliver nutrients [1] and drugs [2], [3]. Unstable messenger RNA molecules have recently been encapsulated in liposomes to form stable vaccines, thus these liposomes are promising drug carriers with practical clinical applications [4], [5]. A drug delivery system (DDS) is required not only to deliver drugs with high efficacy but also to deliver drugs with strong adverse reactions to desired tissues in order to avoid damaging other tissues. Various liposome-based drug delivery methods have been developed, including passive delivery methods with surface modification [6], [7] and active delivery methods using magnetic nanoparticles [8].

Drugs need to be released from the carriers once they reach the desired tissue. Controlled release of drugs involves either passive release through biodegradation or active release using external stimuli. Passive release methods are often individual-specific, making it difficult to achieve stable release times. Therefore, methods for on-demand controlled release using external stimuli have been reported, for example using external stimuli [9], heat [10], light [11], magnetism [12], and ultrasound [13]. This study focused on ultrasound, which has both excellent directionality and transparency. To release drugs from liposomes through factors such as the collapse of cavitation that occurs around or inside the liposomes. Releasing drugs from liposomes using ultrasound occurs through the formation and collapse of tiny gas particles in the hydrophobic region of the lipid bilayer [14]. However, this method requires high irradiation to disrupt liposomes, which can damage the surrounding tissues. This has prevented the clinical application of ultrasound-based drug release from liposomes. Acoustic-responsive liposomes are therefore required in order to safely release drugs by ultrasound *in vivo*.

**Fig. 1:**
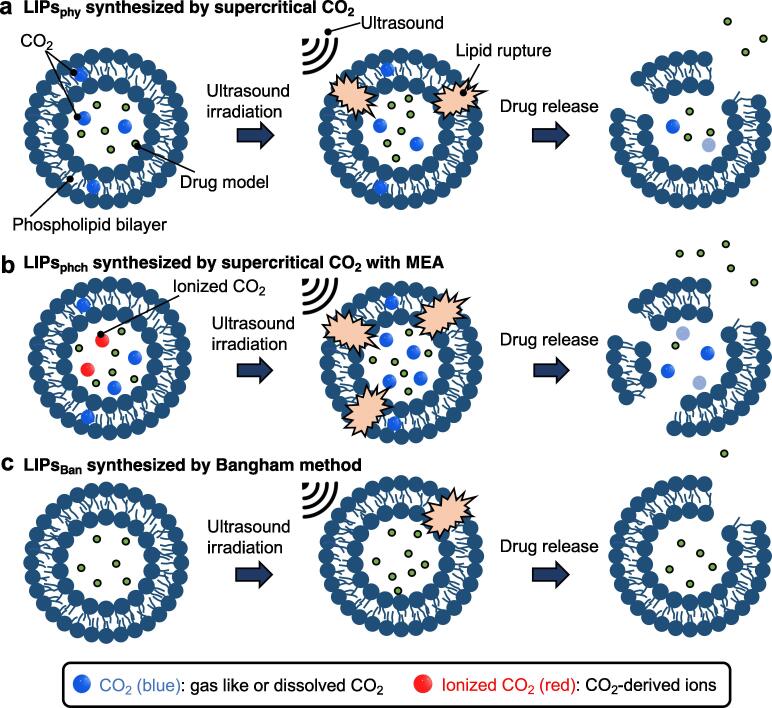
Concept of drug release from liposomes by ultrasound. (a) CO_2_-loaded liposomes physically doped under high pressure CO_2_ (LIPs_phy_) and (b) CO_2_-loaded liposomes chemically doped with ionized CO_2_ by adding monoethanolamine (MEA) to the physically process under high pressure CO_2_ (LIPs_phch_). The water in LIPs_phy_ is filled with CO_2_ molecules. In LIP_phch_, the water is filled with CO_2_ molecules and ionized CO_2_. (c) As a control, liposomes were synthesized using the Bangham method (LIPs_Ban_).

Here, we present nanosized drug carriers with excellent acoustic responsiveness using liposomes synthesized using supercritical CO_2_ (scCO_2_) under high pressure. We prepared CO_2_-loaded liposomes physically doped under high pressure CO_2_ (Fig. 1a) and CO_2_-loaded liposomes chemically doped with ionized CO_2_ by combining monoethanolamine (MEA) with the physical process (Fig. 1b). These synthesized liposomes improved the efficiency of drug release via ultrasonic irradiation compared to drug release from liposomes made using the conventional Bangham method (Fig. 1c). Notably, CO_2_-loaded liposomes synthesized with ionized CO_2_ showed significantly higher acoustic responsiveness.

## 2 Materials and methods

### 2.1 Materials

Egg-derived lecithin (No. 124–05031, Fujifilm Wako Pure Chemicals), cholesterol (No. 034–03002, Fujifilm Wako Pure Chemicals), sodium hydroxide (No. 192–15985, Fujifilm Wako Pure Chemicals), 5(6)-carboxyfluorescein (No. 21877, Sigma-Aldrich), 1 M Tris-HCl (pH 7.5) (No.318–90225, Fujifilm Wako Pure Chemicals), ethanol (No.057–00456, Fujifilm Wako Pure Chemicals), and carbon dioxide (purity > 99.95 %, Fujii Bussan) were used to synthesize fluorescent liposomes that physically load CO_2_. Monoethanolamine (MEA, No. 016–12453, Fujifilm Wako Pure Chemicals) was used to synthesize fluorescent liposomes that chemically load CO_2_, while 1 M Tris-HCl (pH 7.5) was used as a buffer.

Lecithin, cholesterol, sodium hydroxide, and 5(6)-carboxyfluorescein (5,6-CF), were used to synthesize fluorescent liposomes using the Bangham method, with Tris-HCl (1 M, pH 7.5) as the buffer solution. Methanol (No. 131–01826, Fujifilm Wako Pure Chemicals) and acetic acid (No.017–00273, Fujifilm Wako Pure Chemicals) were used to prepare the mobile phase for high-performance liquid chromatography (HPLC) analysis.

### 2.2 Preparation of stock solutions

To synthesize fluorescent liposomes for the drug release experiments, a fluorescent solution of 5,6-CF (excitation at *λ*_ex_ = 490 nm and emission at *λ*_em_ = 520 nm) was prepared. Briefly, 5,6-CF was dissolved in NaOH solution to prepare the solution containing 10 mmol/L of 5,6-CF and 30 mmol/L of NaOH. Next, 1 mol/L Tris-HCl buffer was mixed with ultrapure water to prepare 100 mmol/L Tris buffer solution. Finally, the 5,6-CF solution was mixed with the 100 mmol/L Tris-HCl buffer to prepare a fluorescent solution containing 5 mmol/L of 5,6-CF, 15 mmol/L of NaOH and 50 mmol/L of Tris-HCl. A 50 mmol/L Tris buffer solution, the same concentration as that used for the liposome suspension, was used to wash the liposomes.

### 2.3 Prediction of quantity of CO_2_ loading in synthesized liposomes

After the synthesis of LIPs_phy_ (Fig. 1a) and LIPs_phch_ (Fig. 1b), CO_2_ can be included in its inner aqueous phase and lipid bilayer as gas, dissolved (molecular CO_2_) and ionized states. In the figure, CO_2_ denotes gas-like and dissolved CO_2_ in the aqueous phase or lipid bilayer, and ionized CO_2_ denotes CO_2_-derived ions of carbonate and carbamate species in the aqueous phase. In this work, the loading amount of CO_2_ as dissolved (molecular CO_2_) and ionized states in the aqueous phase was predicted to estimate the thermodynamic potential of CO_2_ loading and to design the synthesis conditions of LIPs_phy_ and LIPs_phch_.

Peng Robison equation [15] and the below mixing rules were used to solve water-CO_2_ phase equilibrium and to calculate the physical loading amount of CO_2_ (corresponding to CO_2_ solubility in water) in LIPs_phy_ and LIPs_phch_.

(1) $$a_{m}=\sum_{i}\sum_{j}x_{i}x_{j}{\left(a_{i}a_{j}\right)}^{1/2}\left(1-k_{\mathrm{ij}}\right)$$

(2) $$b_{m}=\sum_{i}\sum_{j}x_{i}x_{j}b_{\mathrm{ij}}$$

(3) $$b_{\mathrm{ij}}=\left(b_{i}+b_{j}\right)/2$$

where *a*, *b,* and *x* represent attracting force, size factor, and molar fraction, respectively. Subscripts represent pure components *i* and *j*, and the mixture. Phase equilibrium data were obtained from published literature [16] and used to determine the binary interaction parameters *k*_ij_ at the synthesis temperature.

Additionally, the CO_2_ chemical loading amount was predicted by solving a set of nonlinear equations that include the chemical equilibria (Table S1), the charge balance, and the mass balance. Chemical equilibrium constants of the species were obtained from previous studies [17], [18], [19], [20]. In this calculation, the CO_2_ concentration in water represents CO_2_ solubility (result of the calculation in the previous paragraph). The Debye-Huckel equation was used to estimate the activity coefficient of ionic species, where the water activity was regarded as unity. The set of nonlinear equations was solved simultaneously to predict CO_2_ chemical loading amount *C*_Ionized CO2_, which was defined as the total concentration of ionized CO_2_ species using the following equation.

(4) $$C_{Ionized\;CO2}=\left[{\mathrm{CO}}_{3}^{2-}\right]+\left[{\mathrm{HCO}}_{3}^{-}\right]+\left[{\mathrm{TrisCOO}}^{-}\right]+\left[{\mathrm{RNHCOO}}^{-}\right]$$

where *R* denotes the organic part of MEA, apart from the amine group.

### 2.4 Synthesis of CO_2_-loaded liposomes

Liposomes that were physically loaded with CO_2_ (LIPs_phy_) were synthesized using scCO_2_ in a microfluid process as previously described [21]. Briefly, lecithin and cholesterol were dissolved in ethanol to prepare a lipid solution containing lecithin and cholesterol concentrations of 0.150 wt% and 0.105 wt%, respectively, and a weight ratio of 20:14. Liquefied CO_2_ and the lipid solution were supplied using HPLC pumps (PU4386 and PU-4180; JASCO Co., ltd.) and mixed in the *T*-typed junction to dissolve the lipids in scCO_2_. The flow rates of CO_2_ and lipid solution were set to 1000 mL min^−1^ (under atmosphere) and 0.26 mL min^−1^, respectively. The 5,6-CF solution was supplied at a flow rate of 0.01 mL min^−1^ via the HPLC pump (PU-4180, JASCO Co. ltd.) and mixed with scCO_2_ phase in the micro swirl mixer (4-way Swirl Mixer 4–1/16YSM-0.8–0.5-S; Sugiyama Shoji Co., ltd) to form a Water/scCO_2_ (W/scCO_2_) emulsion, with the phospholipid of lecithin acting as a surfactant to stabilize water droplets. Aqueous Tris-HCl solution (50 mmol/L) was supplied at a flow rate of 0.10 mL min^−1^ via a HPLC pump (PU-4180, JASCO Co., ltd.) and mixed with W/scCO_2_ emulsion to form the slug flow. In the slug flow composed of W/scCO_2_ emulsion and water phase, water droplets in the emulsion are captured at the two-phase boundary, resulting in formation of liposomes that physically load CO_2_. This phenomenon proceeds under high pressure CO_2_, which leads to the CO_2_-loading in liposomes. The liposome solution phase and scCO_2_ phase flow into the high-pressure cell where they are separated. The liposome solution phase and scCO_2_ phase are continuously depressurized and extracted from the high-pressure cell through the metering valves at the exits, respectively. The system temperature and pressure were controlled at 313.2 K and 10.0 MPa (corresponding to supercritical CO_2_ conditions) using a thermostat bath and a back-pressure regulator (26–1762-24, TESCOM Co., ltd.). All tubes in the microfluid process have a SUS316 body with an inner diameter of 500 μm. To synthesize CO_2_-loaded liposomes that are chemically doped with ionized CO_2_ by adding monoethanolamine (MEA) to the supercritical CO_2_ process (LIPs_phch_), MEA was dissolved in 5,6-CF aqueous solution (50–200 mmol/L) supplied to the microfluidic system. Other procedures and conditions were similar to those used to synthesize LIPs_phy_.

The prepared 15 mL liposome suspension was dialyzed against 135 mL of the buffer using Float-A-Lyzer G2 (G235073, Spectrum Laboratories, Inc.). After synthesis, the samples were subjected to three rounds of dialysis to eliminate non-encapsulated 5,6-CF, cholesterol, MEA and CO_2_ species. The liposome solutions were then stored at 4 °C before characterization and drug release experiments.

### 2.5 Synthesis of Bangham liposomes

For the control group, Bangham liposomes (LIPs_Ban_) without CO_2_ loading were formed by modifying the Bangham method (thin-film rehydration method) [22], [23] to make it suitable for use with the materials used in this study. To utilize the same materials as CO_2_-loaded liposomes, LIPs_Ban_ were prepared using lecithin and cholesterol (weight ratio of 20:14). Hence, two stock solutions of lecithin and cholesterol at a concentration of 10 mg/mL were mixed with chloroform. A thin film of mixed lipid of lecithin and cholesterol was obtained by air drying for approximately 7 min until the chloroform had completely evaporated. Next, 1 mL of 5,6-CF solution was added to the thin film of dried lipids, and the mixture was sonicated in an ultrasonic cleaner (USC-100Z38S-22, IWAKI Co., ltd) for 15 min. The resulting liposomes were filtered to a homogeneous diameter using an extruder (Avanti Mini Extruder, Avanti® Co., ltd) and a filter (WHA10417004, Whatman® Nuclepore Track-Etch Membrane, Whatman® Co., ltd) with a 0.4 µm pore size. The mixed solution was centrifuged at 4000 × *g* for 30 min using a microcentrifuge (Model 3700, KUBOTA Co., ltd) and Amicon Ultra centrifugal filter units (No. UFC810008, fractional molecular weight 100 kDa, MERCK) and washed with Tris buffer solution. The filtrate was washed by repeated centrifugation until the fluorescent color was no longer visible in a dark room under short-wavelength light. The liposome solution was stored at 4 °C prior to characterization and drug release experiments.

### 2.6 Characterization of liposomes

The liposome size and size distribution were measured at 25 °C using particle tracking analysis technique using the ViewSizer 3000 (HORIBA Instruments Inc). The zeta potential of liposomes was measured using nano Partica SZ-100-Z (HORIBA ltd). Liposome morphology was visualized using transmission electron microscopy (H-7650 Zero. A, Hitachi, ltd) operated at 60 kV and negative staining method (Fig. S1). For the observation, the liposome solution was transferred onto 200-mesh copper grids with an organic membrane (Nisshin EM Co., ltd.). The analyses and observations were performed using the stored liposome solutions after washing with either dialysis or centrifugation.

Encapsulation efficiency (EE) was defined as the proportion of total 5,6-CF used in the synthesis experiment that was encapsulated in the liposomes. To evaluate EE, the liposome solution was dialyzed once prior to washing, using the buffer and Float-A-Lyzer G2 (G235073, Spectrum Laboratories, Inc.). To determine the concentration of 5,6-CF, the dialyzed solution was analyzed using HPLC. The system consists of a HPLC pump (PU-4580), an oven (CO-4060), a column (Unifinepak C18) and a UV–vis detector (UV-4570) with detection wavelength set to 492 nm. The mobile phase was composed of water, methanol, and acetic acid with a weight ratio of 70:30:0.2.

### 2.7 Experimental setup for ultrasonic irradiation

**Fig. 2:**
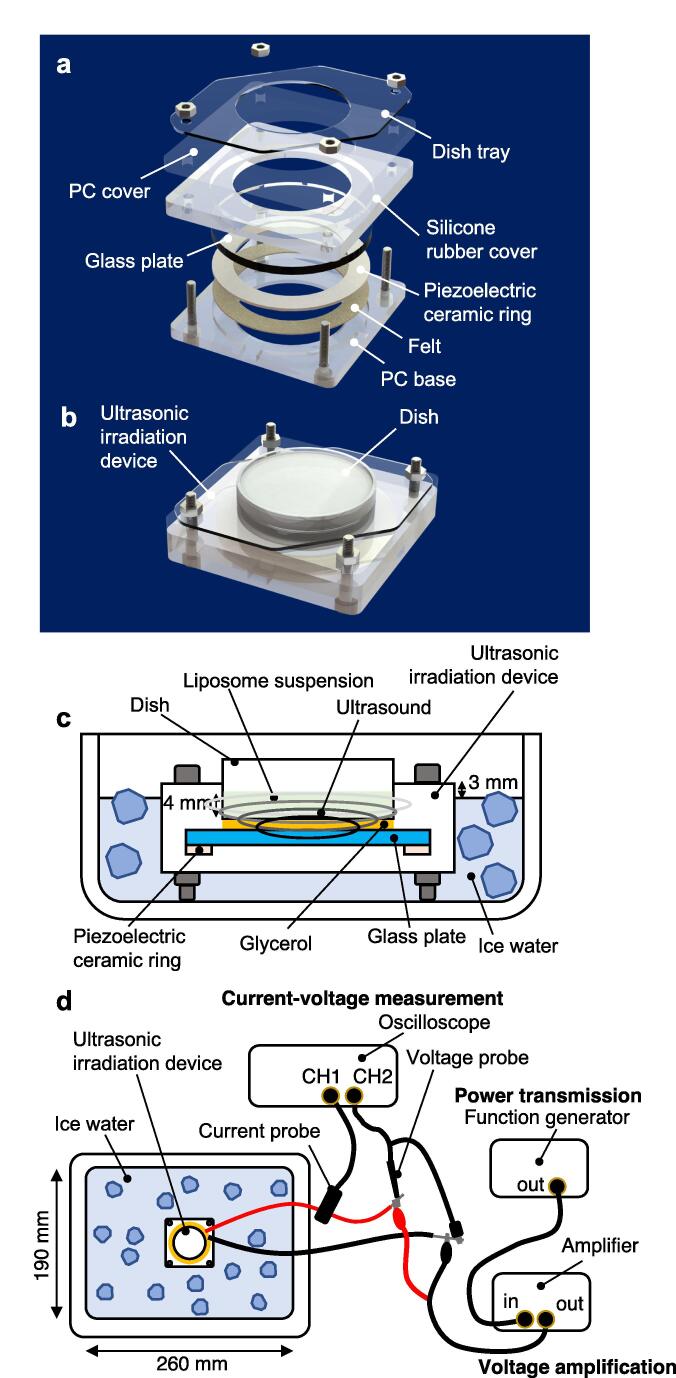
Experimental setup of the ultrasonic irradiation device. (a, b) Ultrasonic irradiation device, showing (a) its components and (b) how the dish is mounted in the device. (c) Ice water was used to cool the system during ultrasonic irradiation. (d) Applying current to the ultrasonic irradiation devices and measuring current and voltage.

A previously described ultrasonic irradiation device [24] was modified and used to degrade the liposomes (Fig. 2a). The space between the glass part of the ultrasonic irradiation device and the dish (35 mm in diameter, 171099, Thermo Fisher Scientific) was filled with glycerol (075–00616, FUJIFILM Wako Pure Chemical Corporation) to enhance the transmission of vibrations (Fig. 2b). A 4-mL volume of liposome was suspended in the dish. The liquid surface of the liposome suspension in the dish was 4 mm above the surface of the glass part of the ultrasonic irradiation device (Fig. 2c). The bottom of the ultrasonic irradiation device was kept in ice in a styrofoam box (260 mm × 190 mm × 150 mm) to prevent the ultrasonic irradiation device from heating. Taking into account its use *in vivo*, a higher frequency was used than in the previous study [24], which is less prone to occur cavitation. Furthermore, a middle-range frequency was used because high ultrasonic frequencies lead to thermal effects [25]. Ultrasound at around 250 kHz has been used for transdermal administration [26], [27] and is also expected to be used for cancer treatment [28] and neuromodulation of the brain [29]. To generate the ultrasound, a function generator (WF1947, NF Co., ltd) transmitted a continuous wave of voltage with a frequency of 237 ± 2 kHz and 1.57 ± 0.02 MHz. The voltage was amplified (HSA4051, NF Co., ltd) and applied to the ultrasound irradiation device (Fig. 2d). The voltage and current of the ultrasound irradiation device were measured using an oscilloscope (tbs2000, Tektronix), and the voltage was adjusted to keep the current constant at 1.5 A, 1.0 A, 0.5 A, 0.25 A and 0.10 A. These ultrasonic irradiation conditions were used to degrade the liposomes two weeks after the synthesis. The temperature of the solution in the dish was measured every minute by measuring the inside of the styrofoam container using a thermistor (TM-300, AS ONE).

The vibration and acoustic pressure generated inside the dish during ultrasonic irradiation were examined. First, vibration amplitudes were measured using a laser Doppler vibrometer (VibroFlex Connect VFX-F-110, Polytec). Then, a fiber-optic acoustic pressure probe [30], [31] and needle hydrophone (HNR-0500, Onda Corporation) were used to measure the acoustic pressure of kHz and MHz bands, respectively. Under the same ultrasonic irradiation conditions as those used for the ultrasonic irradiation of liposomes, the tip of the optical fiber was placed approximately 3.5 mm from the bottom of the dish and the acoustic pressure measured at 7 points spaced 5 mm apart from the center of the dish (35 mm).

### 2.8 Measurement of drug models released from liposomes

To measure the amount of 5,6-CF released from liposomes, peak fluorescence values were measured using the F-7000 spectrophotometer (Hitachi). The ultrasound release efficiency of the liposome drug model (*I*_us_/*I*_all_) was evaluated by dividing the amount of fluorescence released by ultrasound from the liposomes, *I*_us_, by the total amount of fluorescence contained in the liposomes, *I*_all_. To measure *I*_all_, liposomes were placed in 2-mL screw-tube bottles and heated on a hot plate at 90 °C for 120 min, until the entire liposome structure was completely degraded. The heated solution was then centrifuged at 4000 × *g* for 30 min to remove degraded lipid membranes. The fluorescence released from the liposomes was measured to obtain *I*_all_. For *I*_us_, un-degraded liposomes and ultrasound-degraded lipid membranes were removed by centrifuging the ultrasound-irradiated liposomes at 4000 × *g* for 30 min. The fluorescence emitted by the filtrate was measured to represent the fluorescence released by ultrasound irradiation.

### 2.9 Statistical analysis

All experiments were conducted at least in triplicate. Data were analyzed using analysis of variance (ANOVA) with Ryan’s multiple comparison test. Results are expressed as mean ± standard deviation (S.D.). Statistical significance was set at *p < 0.05 and **p < 0.01.

## 3 Results

### 3.1 Characterization of liposomes

Differences in liposome properties, for example size and zeta potential, may affect their stability and effect of sonication on the degradation. To eliminate these effects, liposomes with unimodal size distribution were synthesized, and the mean size was maintained at 214–356 nm (Table 1). The zeta potential was maintained at −9.3 – −2.4 mV.

**Table 1:** Characteristics of LIPs_phy_, LIPs_phch_, LIPs_Ban_.

Method	MEA conc. (mM)	Mean size ± S.D. (nm)^a^	Size distribution ± S.D. (nm)^a^^,^^b^	Zeta potential ± S.D. (mV)^a^	EE ± S.D. (%)^a^
LIPs_phy_	0	284 ± 5	146 ± 6	−2.4 ± 0.3	31.6 ± 0.3
LIPs_phch_	50	257 ± 2	172 ± 1	−7.4 ± 0.4	39.0 ± 0.0
LIPs_phch_	100	214 ± 6	147 ± 5	−3.5 ± 1.2	22.1 ± 0.9
LIPs_phch_	200	235 ± 0	145 ± 3	−9.3 ± 0.9	46.3 ± 0.0
LIPs_Ban_	0	356 ± 3	236 ± 5	−8.1 ± 0.3	5.3 ± 0.7

MEA, monoethanolamine; EE, encapsulation efficiency.

a Standard deviation is obtained from thrice analyses.

b Size distribution is expressed as the standard deviation of liposome size.

### 3.2 CO_2_ concentration in each liposome

**Fig. 3:**
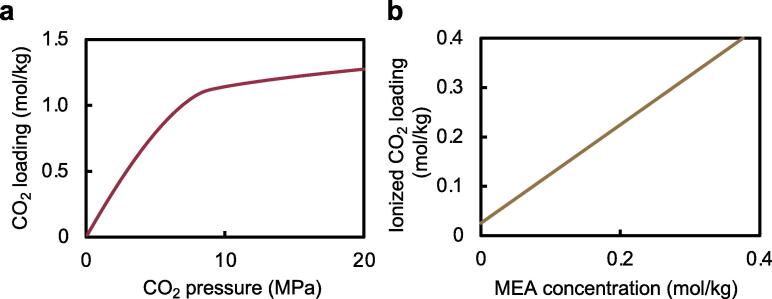
Predicted loading amount of CO_2_ and ionized CO_2_ in LIPs_phy_ and LIPs_phch_. (a) The loading amount of CO_2_ in LIPs_phy_ and LIPs_phch_ is calculated at 40 °C and is independent of MEA (monoethanolamine) concentration. (b) The loading amounts of ionized CO_2_ in LIPs_phy_ and LIPs_phch_ are calculated at the synthesis condition (40 °C, 10.0 MPa) in the presence of various MEA concentrations. The value at 0 mol/kg of MEA denotes LIPs_phy._

The loading amount of CO_2_ in LIPs_phy_ was predicted by solving the phase equilibra for water + CO_2_ system. The equilibrium approximation is appropriate since CO_2_-loaded liposomes are synthesized via W/scCO_2_ emulsion, which means that mass transfer of CO_2_ into water droplets is rapid due to the large contact area. The loading amount of CO_2_ in LIPs_phy_ increased as pressure increased (Fig. 3a), reaching 1.15 mol/kg under the synthesis condition (10 MPa, 40 °C). However, physically loaded CO_2_ in LIPs_phy_ may permeate the phospholipid bilayer due to its small molecular size and nonpolar property, reducing the amount of physically loaded CO_2_ in the liposome over time. CO_2_-loaded liposomes chemically doped with ionized CO_2_ were also synthesized by adding MEA, which has excellent CO_2_ absorption properties [18], [32], to the scCO_2_ process (LIPs_phch_). The ionized CO_2_ species do not permeate the phospholipid bilayer easily due to increased molecular size [33], and the electric repulsion force [34] between negatively charged CO_2_ species and negatively charged liposome surfaces (Table 1). Additionally, the amounts of ionized CO_2_ loaded at various MEA concentrations (Fig. 3b) were predicted by solving the chemical equilibrium of aqueous solution under scCO_2_ (10 MPa, 40 °C). The approximation of chemical equilibrium is appropriate since dissolved CO_2_ immediately reacts with amine species (amine + water + CO_2_) [18]. From this calculation, the amount of ionized CO_2_ species loaded can be controlled by varying MEA concentrations. The physical CO_2_ loading amount was constant at 1.15 mol/kg under the synthesis conditions (10 MPa, 40 °C), regardless of the MEA concentration.

### 3.3 Temperature of ultrasonic irradiation

**Fig. 4:**
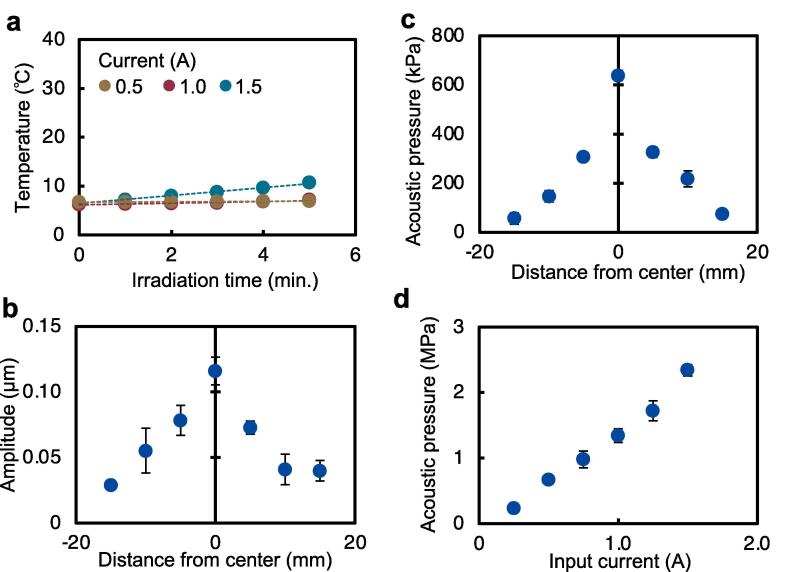
Vibration characteristics of the ultrasonic irradiation device. (a) Changes in the temperature of the solution in the dish were measured using a thermistor. (b) The amplitude distribution of the dish following ultrasound irradiation was measured using a laser Doppler vibrometer. (c, d) The acoustic pressure in the dish was measured using a fiber-optic acoustic pressure probe. (c) The distribution of acoustic pressure and (d) the relationship between current and acoustic pressure are shown. (*n* = 3, mean ± S.D.).

The increase in temperature is caused by prolonged ultrasound irradiation [25]. Since liposomes are sensitive to heat, they can degrade at temperatures above 40 °C. To determine the effect of ultrasound, the effect of temperature needs to be negligible. To evaluate the thermal factor, the temperature inside the dish was measured (Fig. 4a). The results showed that cooling with ice hardly increased the temperature, meaning that the temperature was not far from 40 °C, required for liposome degradation.

### 3.4 Vibration and acoustic pressure of ultrasonic irradiation

To evaluate the output of ultrasound on liposomes, the vibration amplitude on the surface of the dish, and the acoustic pressure generated in the solution in the dish were measured. At first, the distribution of vibration amplitude and acoustic pressure were measured at an applied current of 0.5 A. The measured distribution of vibration amplitude (Fig. 4b) shows that the vibration is highest at the center. Measuring the acoustic pressure distribution also showed that acoustic pressure was highest at the center (Fig. 4c). Next, acoustic pressure was measured at the center of the dish to determine its relationship with input current (Fig. 4d). The acoustic pressure was shown to increase as the applied current.

### 3.5 Drug release from Bangham liposomes and CO_2_-loaded liposomes with and without MEA

**Fig. 5:**
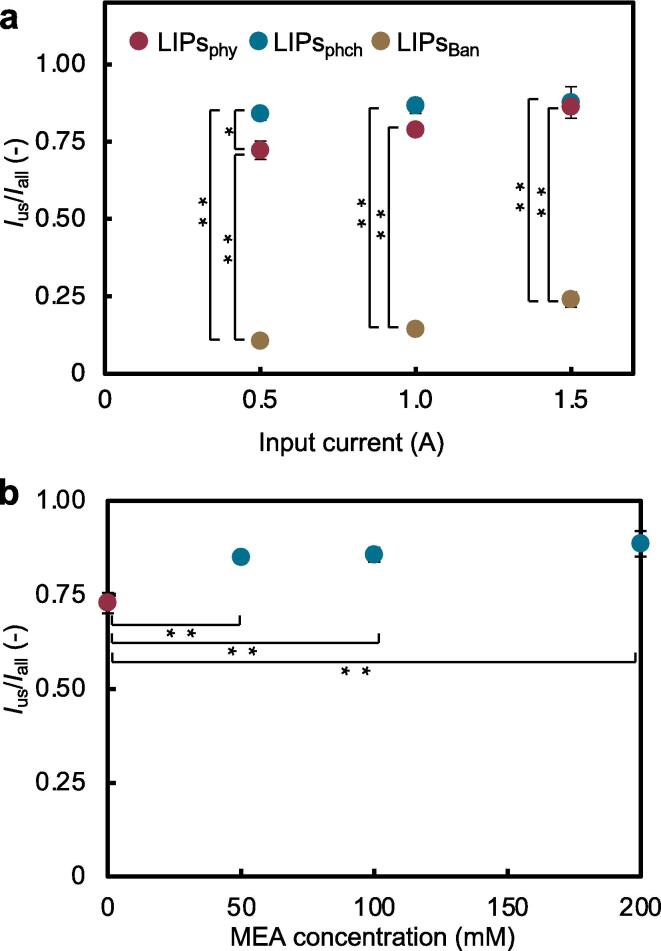
The release efficiency (*I*_us_/*I*_all_) of the model drug from each liposome by ultrasound. (a) Release efficiency of the model drug in LIPs_phy_, LIPs_phch_, and LIPs_Ban_ by irradiation using various output ultrasound currents. (b) Release efficiency of drug model in LIPs_phch_ synthesized with different concentrations of MEA using ultrasonic irradiation. The acoustic pressure of the irradiated ultrasonic waves was 0.67 MPa. (*n* = 3, mean ± S.D., *p< 0.05, **p < 0.01, ANOVA).

The quantities of fluorescence released by ultrasonic irradiation of the LIPs_phy_, LIPs_phch_, and LIPs_Ban_ were measured to determine the release efficiency (*I*_us_/*I*_all_) (Fig. 5a). The amount of released fluorescence was observed at the peak of the fluorescence intensity. The release efficiencies of LIPs_phy_ were 6.83, 5.50, and 3.60 times higher than those of LIPs_Ban_ at applied currents of 0.5 A, 1.0 A, and 1.5 A of ultrasonic irradiation, respectively. The release efficiencies of LIPs_phch_ were 7.94, 6.04, and 3.66 times higher than those of LIPs_Ban_ at applied currents of 0.5 A, 1.0 A, and 1.5 A of ultrasonic irradiation, respectively. Finally, comparing LIPs_phy_ with LIPs_phch_ showed that liposomes doped with MEA (LIPs_phch_) were significantly more efficient at an applied current of 0.5 A of ultrasonic irradiation compared with those not doped (LIPs_phy_). At an applied current of 1.5 A of ultrasound irradiation, LIPs_phy_ and LIPs_phch_ had almost similar release efficiencies. In our experiments, *I*_us_/*I*_all_ peaked at ∼ 0.9, indicating that the amount of release is saturated at a high applied current. Since the materials used for liposome synthesis (lecithin: cholesterol = 20:14) and the conditions of sonication were the same for the three liposomes, these results suggest that CO_2_ inclusion in liposomes improves the efficiency of liposome degradation by ultrasound.

### 3.6 Drug release from CO_2_-loaded liposomes synthesized using different MEA concentrations

To further evaluate the ultrasound-sensitive effect of MEA, the release efficiency of fluorescence was examined by varying the quantity of added MEA (Fig. 5b). Note that 0 mM MEA (=LIPs_phy_) is shown as a control. The mean release efficiency increased with increasing MEA concentration. However, no significant differences were observed, showing that the dependence on the amount of MEA was not observed in our study. This suggests that adding 50–200 mmol/L of MEA enhances the sustained release of drugs from liposomes by ultrasound.

### 3.7 Effects of low power and high frequency

**Fig. 6:**
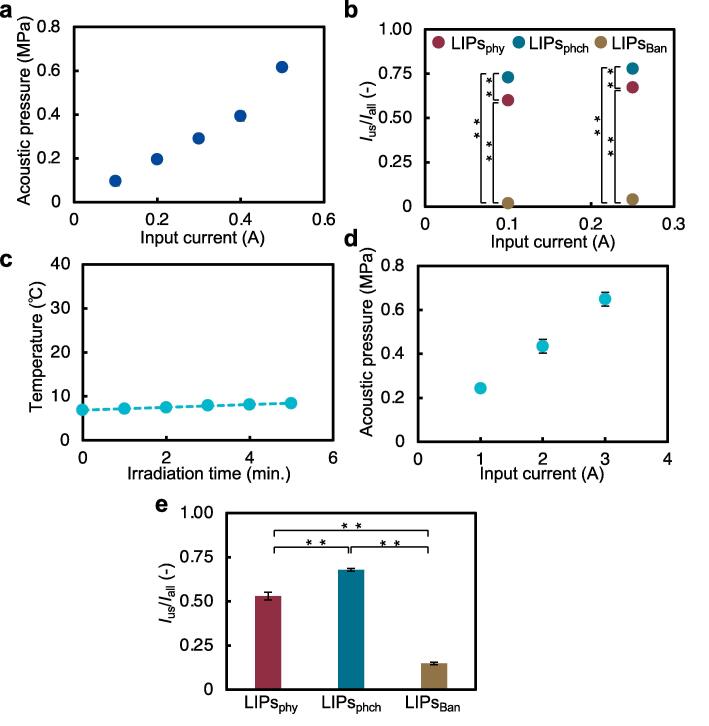
Effects of low power and high frequency on drug sustained release from liposomes. (a,b) Drug release from liposomes at a frequency of 237 kHz with low applied voltage. (a) The relationship between current and acoustic pressure with low applied voltage is shown. (b) Release efficiency of the model drug in LIPs_phy_, LIPs_phch_, and LIPs_Ban_ is irradiated with low-power ultrasound at a frequency of 237 kHz. (c–e) Drug release from liposomes at a frequency of 1.57 MHz. (c) Changes in the temperature of the solution in the dish were measured using a thermistor. (d) The relationship between current and acoustic pressure at a high frequency is shown. (e) The release efficiency of the model drug in LIPs_phy_, LIPs_phch_, and LIPs_Ban_ is irradiated at a high frequency. The acoustic pressure of the irradiated ultrasonic waves was 0.24 MPa (*n* = 3, mean ± S.D., **p < 0.01, ANOVA).

As described above, CO_2_-loaded liposomes have superior acoustic responsiveness compared to conventional liposomes. To further discuss safety, *I*_us_/*I*_all_ at lower power (applied current: 0.25 and 0.1 A) was evaluated. At first, acoustic pressure at a low applied current was measured to evaluate the acoustic intensity (Fig. 6a). The results of the release efficiency (Fig. 6b) show that LIPs_Ban_ hardly released the drug at low power, while LIPs_phy_ (*I*_us_/*I*_all_ = 0.60 at 0.1 A and *I*_us_/*I*_all_ = 0.67 at 0.25 A) and LIPs_phch_ (*I*_us_/*I*_all_ = 0.73 at 0.1 A and *I*_us_/*I*_all_ = 0.78 at 0.25 A) released the drug.

Furthermore, drug release using ultrasound at several MHz, which is commonly used in medical devices, was demonstrated. The relationship between applied current and acoustic pressure (Fig. 6c) was evaluated by using the resonant frequency (=1.57 MHz) of the ultrasound irradiation device. In order to match the acoustic pressure (= ∼0.24 MPa) at low power (237 kHz, 0.25 A), the liposomes were irradiated with MHz ultrasound at an applied current of 1.0 A. *I*_us_/*I*_all_ of LIPs_phy_ (=0.53) and LIPs_phch_ (=0.68) were significantly higher than those of LIPs_Ban_ (=0.15), even when irradiated with MHz ultrasound.

## 4 Discussion

We synthesized liposomes with higher acoustic sensitivity than conventional liposomes and demonstrated their drug release efficiency. When administering liposomes using ultrasound as therapies, the risks posed by the ultrasound to the body should be taken into consideration. The spatial peak-temporal average acoustic intensity (*I*_APTA_) [35] is used to evaluate the safety of ultrasounds. Note that, assuming a free field around the liposome, the acoustic intensity, *I*, is

(5) $$I=\frac{{\left(p_{pp}/2\right)}^{2}}{\rho c}$$

where *p*_pp_, *ρ* and *c* present peak-to-peak acoustic pressure, density and sound velocity. At a frequency of 237 kHz and low power (=0.25 A and 0.01 A), the *I*_APTA_ was 569 mW/cm^2^ and 103 mW/cm^2^, respectively. At a frequency of 1.57 MHz, the *I*_APTA_ was 619 mW/cm^2^. An *I*_APTA_ of 720 mW/cm^2^ or lower guarantees safety *in vivo* [35], thus low-power 237 kHz and MHz ultrasound irradiation conditions are safe. Another safety factor to consider is the heat generated by ultrasounds. In our experiments, liposomes were placed on ice baths and irradiated with ultrasound for a short time (5 min) to prevent them being disrupted by the heat. When using ultrasound in the body, heat generation can be suppressed by changing the ultrasound to pulsed waves and providing a cooling period [25]. Other ways to prevent heat generation include focusing the ultrasound waves using high-intensity focused ultrasound (HIFU) [36], which suppresses the ultrasound output in areas other than the target tissue. Cavitation collapse also generates heat, occasionally damaging biological tissue [37]. In our study, the nuclei are nano-sized liposomes, which are smaller than conventional micro-sized cavitation nuclei, implying that tissue damage is minimal.

When using acoustic-responsive nanomaterials as drug carriers, their stability should be taken into consideration. Phase-changed droplet is a similar nano-sized drug carrier with an acoustic response, where the ultrasound induces the vaporization of oil droplets in water, leading to drug release [38]. Although this carrier shows good ultrasound response, the size of nanodroplets easily increases within several days or weeks of storage time, which is caused by the coalescence [39] and diffusion phenomena [40]. Such size variation, namely low stability, of nanodroplets changes the vaporization threshold by ultrasound [41], which could make it difficult to stably release the drug. Whereas CO_2_-loaded liposome showed improved release efficiency and high reproducibility even after storing the liposome for two weeks, suggesting that it has high stability and allows stable drug release by ultrasound. Moreover, this liposome-based drug carrier is a sphere composed of a bilayer of amphiphilic lipids, which allows for the loading of a wide variety of drug types [42]. Specifically, hydrophobic drugs can be inserted into the liposome membrane, and hydrophilic drugs can be loaded in the inner aqueous phase. In addition, high-molecular compounds (functional nucleic acids and various proteins) can be included. Thus, the acoustic response of the drug carriers with high flexibility has been achieved.

In this study, the drug release efficiency by ultrasound was significantly improved by loading CO_2_ into liposomes. Several factors are considered to be responsible for the disruption mechanism of liposomes. Since the liposomes are loaded with CO_2_, their characteristics seem to be similar to those of ultrasound-responsive droplets [38]. The gas in the liquid droplet acts as cavitation nuclei and induces the drug release, this behavior has been reported. The excellent acoustic response demonstrated in this study can also be attributed to CO_2_ as a cavitation nucleus. The results of drug release with two frequencies at the same acoustic pressure (Fig. 6b,e) shows that the drug release efficiency increased with 237 kHz ultrasound compared to 1.57 MHz ultrasound. Since cavitation is more likely to occur at kHz than at MHz [43], this result enhances the hypothesis that cavitation is a factor in the disruption of CO_2_-loaded liposomes.

In addition, acoustic streaming caused by cavitation [44] is also a candidate for disruption factors. The presence of CO_2_ gas in liposomes can promote acoustic streaming due to nonlinearity, leading finally to transient structural deformation of liposomes due to acoustic streaming. Besides them, the acoustic field within the dish is complicated [24], and standing waves and sloshing also occur at high power. These factors may contribute to liposome disruption. Although these interactive factors are possible, the phenomena occurring around liposomes are not easy to observe due to their nano size, and it is difficult to simply rule out each of these factors.

In this research on the acoustic response of CO_2_-loaded liposomes, MEA was used to investigate the acoustic response of liposomes that are chemically loaded with CO_2_ because MEA has excellent CO_2_ absorption properties and a lot is known about its chemical equilibrium [18], [32]. Although MEA has low biocompatibility, an amino acid with an excess amino group and weakly basic property such as lysine, arginine, glutamine and histidine, can be used to chemically load CO_2_. Additionally, these amine species are widely known to have temperature responsivity for CO_2_ absorption and desorption based on detailed investigations of CO_2_ capture and storage technology [18], [32], [45]. This temperature responsiveness is an advantage for use in DDS. Amine species absorb large amounts of CO_2_ as a state of ionized CO_2_ at low temperatures and desorb CO_2_ molecules at high temperatures [45]. This phenomenon is described as chemical equilibrium shift of the amine + CO_2_ + water system. Additionally, liposomes are typically stored in the refrigerator before being administered to humans, and the temperature difference of about 30 °C is enough to release sufficient CO_2_ molecules from CO_2_ chemical species [32], [45]. Therefore, the temperature responsivity of liposomes that are chemically loaded with CO_2_ can facilitate the in-situ release of CO_2_ molecules in *in vivo* systems, leading to more effective acoustic-responsive drug release.

This technology may have wider application when combined with magnetic liposomes. Recent developments demonstrate the unique property of the magnetoliposomes that encapsulate magnetic iron oxide nanoparticles in the liposome interior. Magnetoliposomes allow for target-selective drug delivery using magnetic navigation [46], [47], followed by drug release triggered by ultrasonic irradiation [48], [49]. Moreover, biodistribution of the magnetoliposomes can be non-invasively measured using magnetic resonance imaging (MRI) because magnetic nanoparticles decrease spin–spin relaxation time (*T*_2_) of the surrounding water protons, providing signal changes in *T*_2_-weighted imaging [50], [51]. Although ultrasound irradiation of magnetic liposomes has already been demonstrated [52], the liposomes themselves are not equipped with ultrasound responsiveness of the kind used in our study. Therefore combining our acoustic-responsive liposomes with magnetic nanoparticles will enable active drug delivery guided by MRI, leading to significantly enhanced therapeutic efficacy.

## 5 Conclusion

The CO_2_-loaded liposomes (LIPs_phy_ and LIPs_phch_) synthesized in this study showed significantly more efficient drug release compared with conventional liposomes (LIPs_Ban_). In particular, release efficiency under ultrasonic irradiation (applied current of 0.25 A) with low risk to the body was 67 % and 77 % with LIPs_phy_ and LIPs_phch_, respectively, compared with 4 % with the LIPs_Ban_. These results indicate that CO_2_-loaded liposomes synthesized under high pressure CO_2_ via a microfluidic process have better acoustic responses than liposomes prepared via conventional methods. In particular, CO_2_-loaded liposomes doped with MEA showed excellent drug-releasing properties. This provides a liposome synthesis technique that enables more efficient on-demand drug release by ultrasound for development of a promising drug carrier in the DDS field.

## CRediT authorship contribution statement

**Yasuhiko Orita:** Conceptualization, Methodology, Investigation, Funding acquisition, Writing – original draft. **Susumu Shimanuki:** Methodology, Validation, Investigation, Writing – original draft. **Satoshi Okada:** Conceptualization, Methodology, Writing – review & editing, Funding acquisition, Resources. **Kentaro Nakamura:** Methodology, Writing – review & editing. **Hiroyuki Nakamura:** Resources, Writing – review & editing, Supervision. **Yoshitaka Kitamoto:** Resources, Writing – review & editing, Supervision. **Yusuke Shimoyama:** Resources, Writing – review & editing, Supervision. **Yuta Kurashina:** Conceptualization, Methodology, Investigation, Funding acquisition, Writing – original draft, Supervision.

## Declaration of Competing Interest

The authors declare that they have no known competing financial interests or personal relationships that could have appeared to influence the work reported in this paper.
