# Supplementary material for: Acoustic-responsive carbon dioxide-loaded liposomes for efficient drug release
Source: Ultrason Sonochem. 2023 Feb 11;94:106326. doi: 10.1016/j.ultsonch.2023.106326 (PMC9958408; doi:10.1016/j.ultsonch.2023.106326)
Supplement: Supplementary data 1 [file mmc1.docx]

**Supplementary Materials**

Supplementary Information for

# Acoustic-responsive carbon dioxide-loaded liposomes for efficient drug release

Yasuhiko Orita^a#^, Susumu Shimanuki^b#^, Satoshi Okada^c^, Kentaro Nakamura^d^, Hiroyuki Nakamura^c^, Yoshitaka Kitamoto^b^, Yusuke Shimoyama^a^, Yuta Kurashina^b,e^*

**Author addresses and affiliations:**

^a^Department of Chemical Science and Engineering, School of Materials and Chemical Technology, Tokyo Institute of Technology, 2-12-1 Ookayama, Meguro-Ku, Tokyo 152-8550, Japan.

^b^Department of Materials Science and Engineering, School of Materials and Chemical Technology, Tokyo Institute of Technology, 4259 Nagatsutacho, Midori-Ku, Yokohama 226-8503, Japan.

^c^Laboratory for Chemistry and Life Science, Institute of Innovative Research, Tokyo Institute of Technology, 4259 Nagatsutacho, Midori- Ku, Yokohama 226-8503, Japan.

^d^Laboratory for Future Interdisciplinary Research of Science and Technology, Institute of Innovative Research, Tokyo Institute of Technology, 4259 Nagatsutacho, Midori- Ku, Yokohama 226-8503, Japan

^e^Department of Mechanical Systems Engineering, Faculty of Engineering, Tokyo University of Agriculture and Technology, 2-24-16, Nakamachi, Koganei-Shi, Tokyo 184-8588, Japan.

^#^These authors contributed equally: Yasuhiko Orita and Susumu Shimanuki.

^*^Corresponding author. E-mail: kurashina@go.tuat.ac.jp

**This PDF file includes:**

Table S1

Figures S1

**Supplementary Table 1** Set of chemical equilibria.

**Supplementary Fig. 1** The typical negative-stain TEM image of liposomes synthesized in the microfluidic process using (a) no MEA and (b) 100 mM MEA.
